# Spontaneous Evisceration, or “Burst Abdomen,” in Patient with Prior Flood Syndrome Surgical Repair

**DOI:** 10.5811/cpcem.31062

**Published:** 2025-01-12

**Authors:** Matthias Barden, Dustin Marinelli, Kirsten Cable

**Affiliations:** * Hi-Desert Medical Center, Department of Emergency Medicine, Joshua Tree, California; † Eisenhower Health Emergency Medicine Residency Program, Rancho Mirage, California; ‡ University of California Riverside School of Medicine, Riverside, California; § Desert Regional Medical Center Emergency Medicine Residency, Palm Springs, California

**Keywords:** case report, images in emergency medicine, burst abdomen, spontaneous evisceration, flood syndrome

## Abstract

**Case Presentation:**

We present an image and discussion of spontaneous evisceration, or “burst abdomen,” from an anterior abdominal wall hernia. A 61-year-old female with prior history of alcoholic cirrhosis and ascites presented to our emergency department with frank evisceration of multiple loops of small bowel from an open anterior abdominal wall dehiscence. Approximately one year prior to this visit she had also been seen in our department for spontaneous rupture of the skin overlying an umbilical hernia and large-volume external leakage of ascites (Flood syndrome^1^). She required surgery to repair the abdominal wall at that time but had subsequently developed a new ventral hernia extending from the umbilicus across a large portion of her left lower abdomen as well as several other postoperative complications. On the day of presentation, she suffered dehiscence of that one-year-old surgical site resulting in spontaneous evisceration of her small bowel. She was transferred to a facility with acute care surgical capabilities where she remained in critical condition.

**Discussion:**

Spontaneous evisceration from abdominal wall dehiscence is a devastating surgical complication. It tends to occur in the immediate postoperative period but has been reported to occur years later. This patient likely suffered from delayed burst abdomen due to multiple comorbidities and postoperative complications.

## CASE PRESENTATION

A 61-year-old female presented to our emergency department (ED) with frank evisceration of multiple loops of small bowel from an open anterior abdominal wall dehiscence of a ventral hernia. About one year prior, she had presented with spontaneous rupture of the peritoneum and skin overlying an umbilical hernia and large-volume external leakage of ascites (spontaneous paracentesis, also known as Flood syndrome).^1^ This is a rare complication of ascites and carries a high morbidity. The mechanism of spontaneous skin rupture is thought to be related to gradual thinning and weakening of the skin overlying umbilical hernias in patients with longstanding tense ascites.^2^ She was managed with primary surgical closure and placement of an indwelling peritoneal drain to avoid reaccumulating of ascitic fluid as the surgical site was healing.^3^

However, her postoperative course after the Flood syndrome repair was complicated. She developed bacterial peritonitis prompting early removal of her indwelling ascites drain. She also developed a larger area of anterior abdominal wall herniation at the site of the prior umbilical hernia that had ruptured. One week prior to presentation she had been admitted at a different facility for incarceration and obstruction of the ventral hernia.

The night prior to presenting to our ED, she had noticed a small, shallow open wound in the surgical scar overlying her hernia. When she awoke on the morning of presentation, she had found that the site was widely open with extruding bowels as pictured in the Image.

Her husband drove her to the ED and helped her inside via our ambulatory entrance. Upon arrival to triage the exposed bowels were covered with sterile, saline-soaked gauze, and she was started on intravenous (IV) fluids and broad-spectrum antibiotics. The general surgeon recommended transfer to another facility for higher level of care. At the receiving facility she was taken emergently to the operating room for exploratory laparotomy and umbilical hernia repair. During the procedure the bowel was reduced, and an absorbable mesh overlay was used to close the fascia. She was managed in the intensive care unit for multiorgan system failure requiring continuous renal replacement therapy and vasopressor support before she unfortunately died one month later.

## DISCUSSION

While intentionally leaving the abdomen open for postoperative management is an increasingly frequent surgical strategy, the unintentional acute postoperative open abdomen is a rare and serious surgical complication.^4^ Spontaneous evisceration from abdominal wall dehiscence (or burst abdomen) tends to occur in the acute postoperative period but has also been reported to occur years later in the setting of large chronic incisional hernias.^5^,^6^

Our patient was about one year out from surgical repair of a prior spontaneous rupture of an umbilical hernia in the setting of cirrhotic ascites, also known as Flood syndrome. Flood syndrome carries a high rate of mortality, and strong evidence is lacking regarding the best technique for its surgical management.^7^ While this patient survived the initial recovery from Flood syndrome, her postoperative course was quite complicated. These complications, as well as her overall general poor health, likely contributed to weakening and eventual rupture of the soft tissue overlying the ventral hernia site.

**CPC-EM Capsule**

What do we already know about this clinical entity?

*Flood syndrome (spontaneous paracentesis) and burst abdomen (spontaneous evisceration) are rare entities with high morbidity and mortality*.

What is the major impact of the image(s)?

*This image depicts the severity of a presentation of burst abdomen that occurred one year after surgical repair of a case of flood syndrome*.

How might this improve emergency medicine practice?

*Awareness of flood syndrome and burst abdomen may help clinicians respond appropriately to these dramatic presentations*.

Emergency department management was limited to covering the exposed bowel in sterile, saline-soaked gauze, initiating broad-spectrum IV antibiotics, and transferring her for higher level of care to a facility with acute care surgical capabilities where she was managed surgically and with critical care life support; however, she ultimately died from this event.

## Figures and Tables

**Image:**
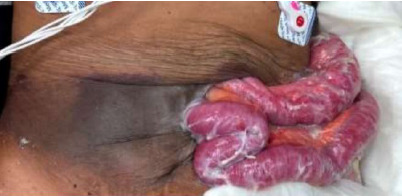
Evisceration of the small bowel from an anterior abdominal wall open wound.
